# Characteristics and Health Risks of Polycyclic Aromatic Hydrocarbons and Nitro-PAHs in Xinxiang, China in 2015 and 2017

**DOI:** 10.3390/ijerph18063017

**Published:** 2021-03-15

**Authors:** Hao Zhang, Lu Yang, Xuan Zhang, Wanli Xing, Yan Wang, Pengchu Bai, Lulu Zhang, Ying Li, Kazuichi Hayakawa, Akira Toriba, Ning Tang

**Affiliations:** 1 Graduate School of Medical Sciences, Kanazawa University, Kakuma-machi, Kanazawa 920-1192, Japan; zhanghao@stu.kanazawa-u.ac.jp (H.Z.); veronicayl@stu.kanazawa-u.ac.jp (L.Y.); zhangxuan@stu.kanazawa-u.ac.jp (X.Z.); xingwanli@stu.kanazawa-u.ac.jp (W.X.); wangyan@stu.kanazawa-u.ac.jp (Y.W.); baipengchu@stu.kanazawa-u.ac.jp (P.B.); 2 Institute of Nature and Environmental Technology, Kanazawa University, Kakuma-machi, Kanazawa 920-1192, Japan; zhang-lulu@se.kanazawa-u.ac.jp (L.Z.); hayakawa@p.kanazawa-u.ac.jp (K.H.); 3 School of Chemistry and Chemical Engineering, Henan Institute of Science and Technology, Xinxiang 453003, China; hnliy0412@126.com; 4 Graduate School of Biomedical Science, Nagasaki University, Bunkyo-machi, Nagasaki 852-8521, Japan; toriba@nagasaki-u.ac.jp; 5 Institute of Medical, Pharmaceutical and Health Sciences, Kanazawa University, Kakuma-machi, Kanazawa 920-1192, Japan

**Keywords:** air pollution, polycyclic aromatic hydrocarbon, nitro-polycyclic aromatic hydrocarbon, Xinxiang, China

## Abstract

Fine particulate matter (PM_2.5_) samples were collected in the summer and winter of 2015 and 2017 in Xinxiang, China. Nine polycyclic aromatic hydrocarbons (PAHs) and three nitro-PAHs (NPAHs) in PM_2.5_ were detected via high-performance liquid chromatography (HPLC). The PAHs concentration in summer and winter decreased from 6.37 ± 1.30 ng/m^3^ and 96.9 ± 69.9 ng/m^3^ to 4.89 ± 2.67 ng/m^3^ and 49.8 ± 43.4 ng/m^3^ from 2015 to 2017. NPAHs decreased in winter (from 1707 ± 708 pg/m^3^ to 1192 ± 1113 pg/m^3^), but increased in summer from 2015 (336 ± 77.2 pg/m^3^) to 2017 (456 ± 312 pg/m^3^). Diagnostic ratios of PAHs indicated that petroleum combustion was the main emission source in summer, and pollutants originating from the combustion of petroleum, coal and biomass dominated in winter. The 2-nitrofluoranthene (2-NFR)/2-nitropyrene (2-NP) ratio in this study demonstrated that the OH radical pathway was the main pathway for the formation of 2-NP and 2-NFR. The mean total benzo[*a*]pyrene-equivalent concentrations (BaP_eq_) and incremental lifetime cancer risk (ILCR) values decreased from 2013 to 2017. The high value of total BaPeq in the winter of 2017 in Xinxiang revealed that a high-risk of cancer remained for residents. The results of this study demonstrate that the decreases in PAHs and NPAHS concentrations from 2015 to 2017. Combined with reducing gaseous pollutants concentration, the reduction in this study might be attributable to emissions reductions by implementing the air pollution control regulations in Xinxiang city in 2016.

## 1. Introduction

As one of the major problems affecting human health, air pollution, such as that related to particulate matter (PM), has received increasing attention [1]. Prolonged exposure to PM has been linked to a risk of both acute and chronic effects, including lung cancer, heart attack, chronic respiratory disease, chronic bronchitis in adults and respiratory infections in children [2,3,4]. Polycyclic aromatic hydrocarbons (PAHs) are some of the most harmful components in PM and are mainly formed via the incomplete combustion of fossil fuels and biomass (e.g., plant material, waste and tobacco) [5]. Incomplete combustion also produces nitro-derivatives of PAHs (NPAHs). However, NPAHs may also be generated via the reaction of PAHs and NO_2_ [6,7]. Both PAHs and NPAHs are harmful, persistent organic pollutants diffusely disseminated in the atmosphere. Compared to PAHs, certain NPAHs were found to be more mutagenic than their parent PAHs [8,9].

Xinxiang, one of the most polluted prefecture-level cities located in Henan Province, China, is located near the provincial capital of Zhengzhou, close to the highly polluted Shanxi Province and near the Jing-Jin-Tang region, which is one of the most important economic circles in China. Approximately 6,198,100 people lived in Xinxiang in 2019, and the population is still exhibiting a continued upward trend [10]. According to the China Statistical Yearbook of 2015, the consumption of raw coal increased by 1.37 million tons to 17.83 million tons, and the number of motor vehicles reached 674,851 in 2015 [11]. Additionally, Xinxiang city achieved an industrial added value of 99.136 billion yuan in 2016, representing an annual increase of 8.3% [12]. It should be noted that with the rapid increase in industry, population and vehicles, Xinxiang also has experienced increasingly severe air pollution [11,13]. To improve the air quality, a pollution prevention action plan, namely, the Xinxiang City Blue Sky Project Action Plan, was initiated in February 2016 [14]. This plan mainly focused on the reduction in emissions originating from the thermal power industry and industrial furnaces. The above plan to decrease the emissions stemming from the thermal power industry included reducing the emissions of main air pollutants originating from coal-fired boilers above 65 steam tons/hour, desulfurizing the coal-fired boilers with a steam capacity higher than 10 tons/hour, and demolition and transformation of coal-fired boilers with a steam capacity of 10 tons/hour and below. Industrial furnaces, such as cement clinkers, lime production lines, foundry plants, etc., were requested to decrease their emissions of dust and air pollutants [15]. According to a report by the Xinxiang County People’s Government, during the plan period, 220 coal-fired boilers were demolished or remodeled, while 77 sand and gravel yards and 46 coal-burning sales points were banned [16]. During only one month in 2016, 10 factories with excessive pollutant emission in Xinxiang were ordered to suspend business for rectification purposes [17]. However, until now, detailed investigations have not been performed on the pollution status and main sources of atmospheric PAHs and NPAHs in Xinxiang.

Thus, in this study, fine PM (PM_2.5_) was collected in Xinxiang in the winter and summer of 2015 and 2017, and nine PAHs and three NPAHs were analyzed. We aim to investigate the changes in PAHs and NPAHs to observe the effect of implementing environmental governance policies in Xinxiang in 2016. The seasonal and yearly conversion levels of PAHs and NPAHs from 2015 to 2017 were discussed to understand the variation of the concentration, composition, emission source and health risk of PAHs and NPAHs since the environmental governance policies started.

## 2. Materials and Methods

### 2.1. PM Sampling

**Figure 1:**
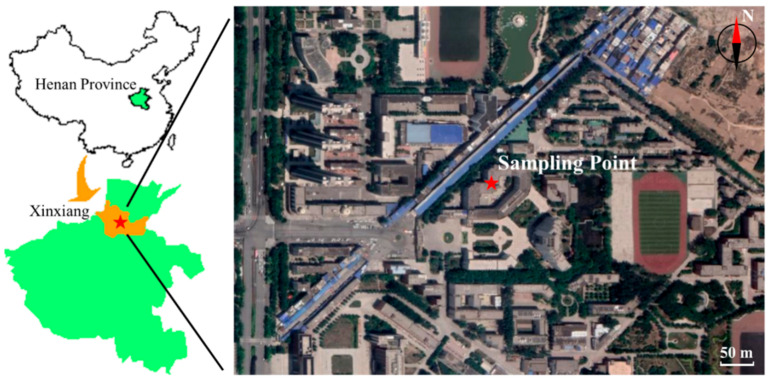
Sampling Point Location Site in Xinxiang, China.

Figure 1 shows the PM_2.5_ sampling point in Xinxiang, China. A sampler was set on the roof (approximately 14 m above ground) of the School of Chemistry and Chemical Engineering of the Henan Institute of Science and Technology (35.3° N, 113.9° E). PM_2.5_ samples were collected with a low-volume sampler purchased from Tokyo Dylek (MCP-3–10251, Tokyo, Japan) and quartz fiber filters (2500QAT-UP, Pall Co., NY, USA) were employed during sampling. The flow rate was 3 L/min. PM_2.5_ samples were collected during four periods: 2015—summer (3 June 2015–15 June 2015; *n* = 6), 2015—winter (26 December 2015–5 January 2016; *n* = 6), 2017—summer (13 July 2017–25 July 2017; *n* = 7) and 2017—winter (5 January 2018–17 January 2018; *n* = 7). All filters were replaced every two days. Each sampling period included 2 weekends (4 days). All samples were well packed and stored at −25 °C before analysis. Data of meteorological conditions and gaseous pollutants in this study were acquired from the China Air Quality Online Monitoring and Analysis Platform (https://www.aqistudy.cn/historydata/ accessed on 20 December 2018). Table S1 summarizes the meteorological conditions.

### 2.2. Sample Analysis

The filters were cut into pieces and were then placed into different flasks. Two internal standards, benzo[*a*]pyrene-*d*_12_ (BaP-*d*_12_, 98%) and pyrene-*d*_10_ (Pyr-*d*_10_, 98%) (Wako Pure Chemicals, Osaka, Japan), were added into each flask. Benzene:ethanol (3:1, *v*/*v*) was added as the extractant, and ultrasonication was adopted for extraction. The extraction was repeated twice. The extracts were filtered and were then washed with NaOH solution (5%, *w*/*v*) and H_2_SO_4_ solution (20%, *v*/*v*) (Wako Pure Chemicals, Osaka, Japan). Followed by the washing of the distilled water. Dimethyl sulfoxide was added, and a rotary evaporator was used to concentrate the extracts to 100 µL. Nine hundred microliter of ethanol was added, and the solutions were then filtered by the 0.45 μm pore size filter membrane (HLC-DISK13, Kanto Chemical CO., Inc., Tokyo, Japan). The filtrates were injected into each vial separately. The samples were then detected via high-performance liquid chromatography (HPLC, Shimadzu Inc., Kyoto, Japan). Nine PAHs, namely, fluoranthene (FR), pyrene (Pyr), benz[*a*]anthracene (BaA), chrysene (Chr), benzo[*b*]fluoranthene (BbF), benzo[*k*]fluoranthene (BkF), benzo[*a*]pyrene (BaP), benzo[*ghi*]perylene (BgPe) and indeno(1,2,3-*cd*)pyrene (IDP) (Supelco Park, Bellefonte, PA, USA), and three NPAHs, namely, 2-nitrofluoranthene (2-NFR) (Chiron AS, Trondheim, Norway), 1-nitropyrenes (1-NP) and 2-nitropyrenes (2-NP) (Aldrich Chemical, Osaka, Japan) were analyzed by HPLC with the fluorescence detection system. All HPLC reagents were stocked from Wako Pure Chemicals (Osaka, Japan).

### 2.3. Quality Control

To confirm whether contamination occurred during sampling prior to analysis, blank filters were analyzed, which revealed no contamination during the process. Good linearity of calibration curves was attained for the standard (*r* > 0.998). The internal standard recoveries of all samples in this study ranged from 75% to 120%. Table S2 presents the limit of determination and range of the calibration curves of PAHs and NPAHs.

### 2.4. Health Risk Assessment

The BaP equivalent concentration (BaP_eq_) and incremental lifetime cancer risk (ILCR) have been commonly applied to determine the potential risk of PAHs to human health [18,19,20]. The equations to determine BaP_eq_ and ILCR are expressed as follows:

(1) $${\mathrm{BaP}}_{\mathrm{eq}}=\sum \left(C_{i}\times TEF_{i}\right)$$

(2) $${\mathrm{ILCR}\;=\;\mathrm{UR}}_{\mathrm{BaP}}\times {\mathrm{BaP}}_{\mathrm{eq}}$$

In Equation (1), *C_i_* and *TEF_i_* are the concentration and reference toxicity equivalence factors, respectively, of a given PAH and NPAH [21,22]. Table S3 lists the *TEF_i_* value of various PAHs and NPAHs.

UR_BaP_ in Equation (2) denotes the unit cancer risk stemming from *BaP*, which is equal to 1.1 × 10^−6^ ng/m^3^ for hamsters and 8.7 × 10^−5^ ng/m^3^ for coke-oven workers [23,24].

### 2.5. Statistical Analysis

SPSS version 25.0 (IBM Corp., Armonk, NY, USA) was applied in this study. The Spearman correlation coefficient was determined to analyze the correlation among PAHs, NPAHs and meteorological conditions. The Paired-sample Wilcoxon test was used to compare the data of PAHs and NPAHs. A *p*-value lower than 0.05 indicated that the results were significant. A *p*-value lower than 0.01 indicated that the results were very significant.

## 3. Results and Discussion

### 3.1. Concentrations and Composition of PAHs and NPAHs

Table 1 and Table S4 summarize the mean and daily concentrations, respectively, of PAHs and NPAHs in summer and winter from 2015 to 2017. The highest mean concentrations of PAHs and NPAHs were both observed in the winter of 2015, at concentrations of 96.9 ± 69.9 ng/m^3^ and 1707 ± 708 pg/m^3^, respectively. The concentration of PAHs during both winter periods was one order of magnitude higher than that during the summer periods. Although the increase in the concentration of NPAHs from summer to winter was much smaller than that in the concentration of PAHs, the NPAHs concentration in winter was still much higher than that in summer. The reason may be residential heating in winter, whereby a large amount of solid fuel is combusted [6]. In addition to changes in emission sources, the relatively strong photochemical degradation reactions in summer increase the degradation rate of PAHs and NPAHs, causing low concentrations in summer [25,26]. In winter, temperature inversion likely occurs in the boundary layer, which also increases the concentrations of PAHs and NPAHs [27].

Compared to previous research in Xinxiang in 2013, which selected a few days in summer and winter for sampling. The mean concentrations of PAHs in 2013 were 18.1 and 145 ng/m^3^ in summer and winter, respectively, and the mean concentrations of NPAHs (only 1-NP and 2-NP) were 318 and 320 pg/m^3^, respectively [28]. The concentrations of both PAHs and NPAHs in Xinxiang in 2013 were higher than those determined in this study in 2015. However, the concentration of PAHs (742.3 ng/m^3^) in Taigu, a nearby city in Shanxi Province, was much higher than that in Xinxiang in 2015 [29]. Compared to Zhengzhou, Beijing and Shanghai during the same period, the concentration of PAHs in 2015 was lower than that in Beijing (7.81 ng/m^3^, summer; 214 ng/m^3^, winter) and Zhengzhou (23 ng/m^3^, summer; 144 ng/m^3^, winter), but much higher than that in Shanghai (1.83 ng/m^3^, summer; 9.51 ng/m^3^, winter) [3,30,31]. The concentration of NPAHs in Xinxiang in 2015 exhibited the same tendency as that of PAHs, which was lower than that in Beijing (5.12 ng/m^3^, winter), but higher than that in Shanghai (103 pg/m^3^, summer; 363 pg/m^3^, winter) [3,30].

As indicated in Table 1, compared with 2015, both the PAH and NPAH concentrations decreased approximately 48% and 30%, respectively, in the winter of 2017. The concentrations of PAHs were similar to those in another study in January 2017 (36.8 ng/m^3^) [32]. The concentration of total PAHs showed a significant difference between 2015 and 2017 (*p* < 0.05). The decrease in the concentration might be due to the strict pollution control policies (e.g., demolition of coal-fired boilers, promotion of clean heating in winter, and control of the vehicle amount and oil quality) implemented in Xinxiang since 2016 [33]. However, in summer, although PAHs were reduced by approximately 23%, NPAHs increased by approximately 35%. Since motor vehicles emit more NPAHs than PAHs, the reason for the increase in summer might be the increasing number of motor vehicles in Xinxiang. From 2014 to 2016, the number of vehicles in Xinxiang increased from approximately 571,000 to 623,000 [34,35]. This finding is consistent with our previous long-term observations in Shenyang [3,6,36].

**Figure 2:**
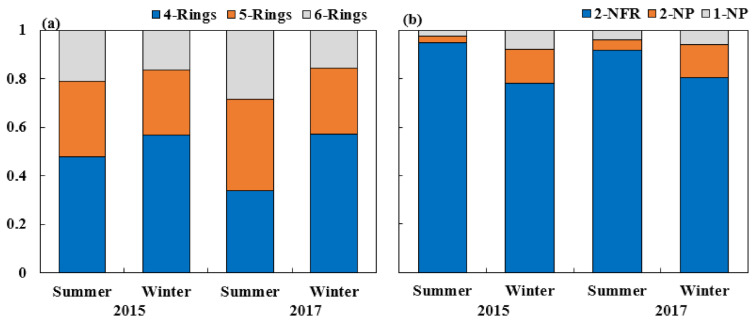
The ratio of each component to the total PAHs (**a**) and NPAHs (**b**) in PM_2.5_ during sampling periods.

In winter, PAHs with four rings dominated the total PAHs, while PAHs with 5- and 6-rings occupied a relatively high proportion of total PAHs in summer. Compared to PAHs with numerous rings, 4-ring PAHs exhibit a relatively high vapor pressure, and they are more easily transition into the particle phase during the winter period [37]. Figure 2a shows the ratio of PAHs with different ring numbers. The mean percentage of PAHs based on the ring number in winter was not significantly different, while in summer, the ratios were 0.21 (6-rings), 0.31 (5-rings), and 0.48 (4-rings) in 2015 and 0.28 (6-rings), 0.38 (5-rings), and 0.34 (4-rings) in 2017. The ratio of 4-ring PAHs in 2017 was lower than that in 2015 in summer. Additionally, the ratio of each NPAH to the total NPAHs was similar in 2015 and 2017 (Figure 2b). 2-NFR accounted for most of the total analyzed NPAHs, followed by 2-NP (78–95% and 3–14%, respectively), and 1-NP (2–8%) accounted for the lowest proportion. The emissions of 5- and 6-ring PAHs and 1-NP originating from vehicles are higher than those originating from coal combustion systems. These results suggested that the main cause of the changes in the composition of PAHs and NPAHs might be the reduction in the contribution rate of PAHs stemming from coal combustion and the increase in PAHs emissions stemming from motor vehicles [38,39,40].

### 3.2. Emission Sources

**Figure 3:**
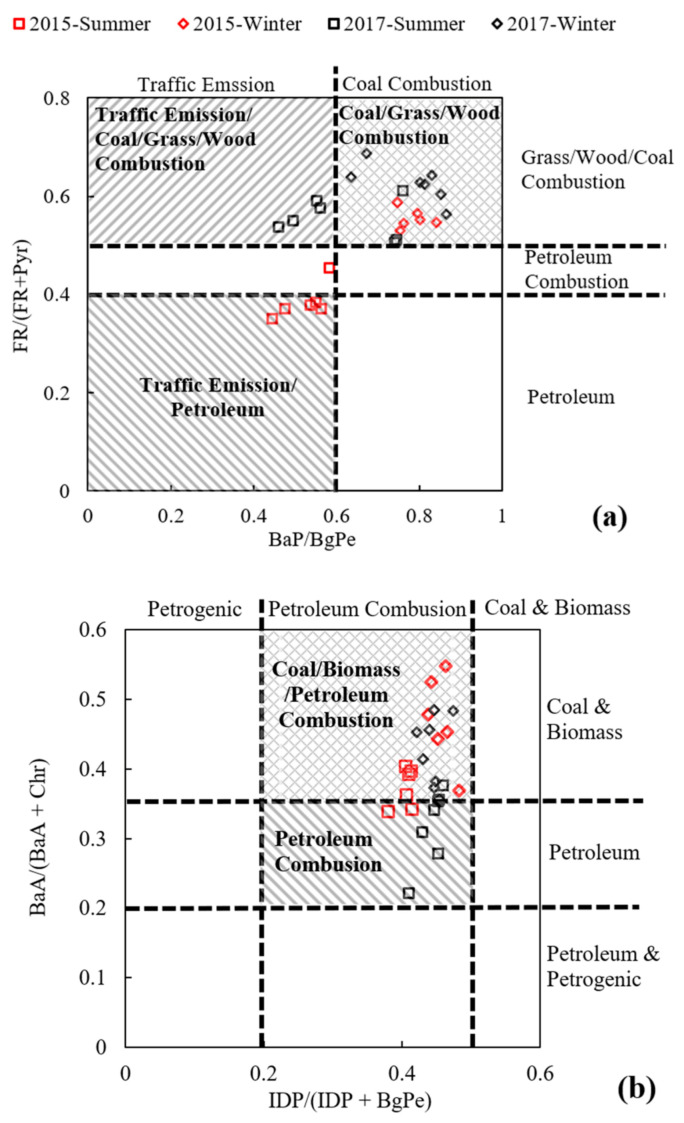

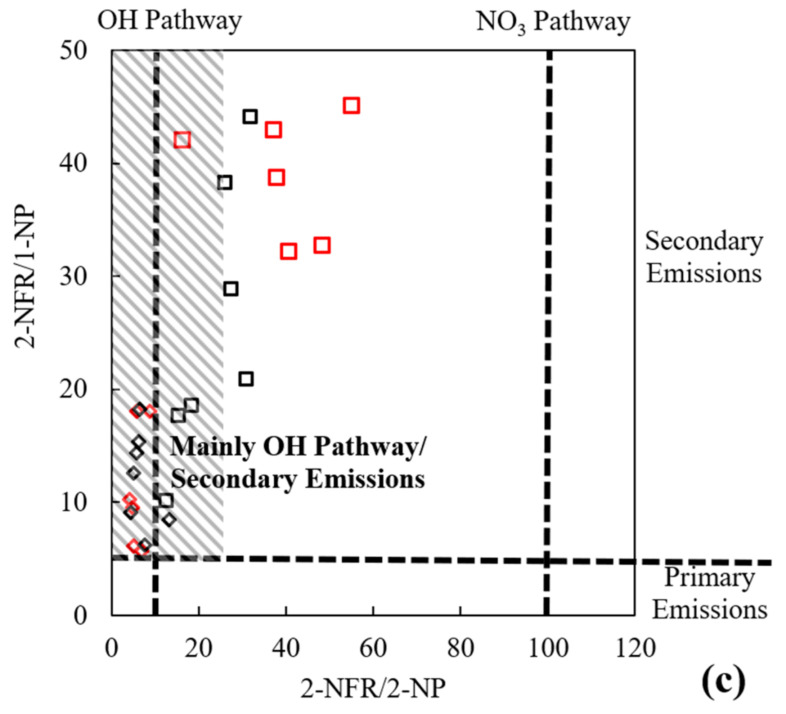
The diagnostic ratios on the sources of PAHs and NPAHs emissions (**a**) BaP/BgPe vs. FR/(FR+Pyr) [45,46] (**b**) IDP/(IDP+BgPe) vs. BaA/(BaA + Chr) [47] (**c**) 2-NFR/2-NP vs. 2-NFR/1-NP [48,49,50,51].

Diagnostic ratios of PAHs have generally been adopted to distinguish the major pollution emission sources [41,42,43,44,45,46]. Figure 3 shows the diagnostic ratios of PAHs and NPAHs in this study. A diagnostic ratio of BaP/BgPe lower than 0.6 indicates traffic emissions [44]. In this study, the value of BaP/BgPe, as shown in Figure 3a, was lower than 0.6 in 2015 in summer (0.52), and it was higher than 0.6 in the summer of 2017 (0.64) and winters of both 2015 and 2017 (0.78 and 0.80). The relatively higher BaP/BgPe value might be demonstrated that traffic emissions imposed a higher effect in summer than in winter. The FR/(FR + Pyr) ratio was adopted to discern the emissions stemming from petrogenic sources (<0.4), petroleum combustion (0.4–0.5) and coal/wood/grass combustion [45]. In this study, the ratio during the summer period of 2015 was in the range of petroleum combustion and petroleum. The ratio during the other periods was higher than 0.5. Combined with BaP/BgPe and FR/(FR + Pyr), traffic emissions exhibited a dominant contribution in summer, but the emissions originating from coal/wood/grass burning cannot be ignored. Figure 3b shows a cross-plot of the diagnostic ratios in regard to the sources of PAH emissions. IDP/(IDP + BgPe) revealed petrogenic sources (<0.2), petroleum combustion (0.2–0.5) and coal and biomass combustion (>0.5) [46]. The BaA/(BaA + Chr) ratio ranged from 0–0.2, and 0.2–0.35 were higher than 0.35, indicating petroleum and petrogenic sources, petroleum combustion and coal and biomass combustion, respectively [46]. These results indicated petroleum combustion in summer (a ratio approximately equal to or lower than 0.35) and mixed petroleum and coal and biomass emissions in winter in Xinxiang (a ratio higher than 0.35). The above results demonstrated petroleum combustion in summer and mixed petroleum and coal and biomass emission in winter in Xinxiang, and the emissions were similar to those reported in other studies in Xinxiang [28].

Figure 3c shows the ratios of NPAHs in this study. In contrast to 1-NP, 2-NP and 2-NFR are only produced in the atmosphere, whereby the corresponding parent-PAHs are nitrated through reactions initiated by free radicals, after which 2-NP and 2-NFR are formed. A 2-NFR/2-NP ratio close to 10 indicates that the formation of NPAHs mainly occurs under the OH radical pathway, and a ratio higher than 100 indicated NPAH formation under the NO_3_ radical pathway in the atmosphere [47,48]. In Xinxiang, although the 2-NFR/2-NP ratio was higher in summer (12.5–55.3) than in winter (4.04–13.2), overall, the ratio was still close to 10. These results indicate that 2-NP and 2-NFP in Xinxiang were mainly formed under the OH radical pathway. The 2-NFR/1-NP ratio is an index used to investigate the aging of particles containing PAHs and NPAHs, and NPAHS with ratios higher than 5 are considered to be primarily formed via secondary formation reactions [49,50]. In this research, the ratios were all higher than 5, especially in summer (10.1–45.0). This result indicates the relatively long occurrence time of PM in the air. It should be noted that the above result of the 2-NFR/1-NP ratio should be further examined via the integrating of the reaction rate of local nitrification, meteorological conditions, and impact of other regions. This may also be another reason for the increase in NPAHs in the summer of 2017, in addition to the increase in motor vehicles.

### 3.3. Meteorological Conditions

Previous studies have shown that the meteorological conditions may influence the diffusion, phase partitioning, generation, accumulation and removal of PAHs [51,52]. Table 2 lists the correlation coefficient among the individual PAHs and NPAHs with meteorological conditions. A significant negative correlation was found between almost all PAHs and NPAHs (*n* = 26, *p* < 0.05) with the temperature, which was caused by the concentration changes in summer and winter mentioned in the previous section. The increase in wind speed may affect the spread of pollutants, but there was no statistically significant correlation in this study. In contrast to other studies in Japan and Vietnam, the correlation of the relative humidity with PAHs and NPAHs in this study was not significant, which was similar to the result in Nanjing, China [51,53,54]. This may occur because the relative humidity was relatively low and varied little during the sampling period. In addition, seasonal correlations between PAHs, NPAHs and the meteorological conditions are shown in Table S5. Several PAHs and NPAHs were significantly correlated with meteorological conditions in summer and winter. However, the humidity and wind speed in this study are relatively average values, and the range of variation is not large during the sampling periods. It can be considered that the changes in meteorological conditions during the study periods had a limited effect on the PAHs and NPAHs.

### 3.4. Gaseous Pollutants

Table 3 presents the concentrations of gaseous pollutants during sampling. Similar to the concentrations of PAHs and NPAHs, most of the gaseous pollutants decreased during the study period. SO_2_ is largely formed through the burning of coal, for example, power generation and heating [55]. SO_2_ exhibited a dramatic decrease by almost one-third in 2017 over 2015 (from 15.66 ± 4.548 part per billion (ppb) to 4.826 ± 0.933 ppb and 34.54 ± 8.740 ppb to 9.842 ± 6.321 ppb for summer and winter, respectively). This decrease might occur to the control of coal combustion emissions. The incomplete combustion of fuel and the oxidation of hydrocarbons in the atmosphere produce CO. In addition, coal-burning and traffic emissions may both increase the concentration of CO [56]. In this study, the CO concentration was halved (from 3.297 ± 1.229 ppb to 1.734 ± 1.129 ppb) from 2015 to 2017 in winter, but it was slightly increased (from 0.730 ± 0.170 ppb to 0.905 ± 0.272 ppb) from 2015 to 2017 in summer. The reason for the decrease in winter might be similar to that for the decrease in SO_2_, while in summer, the increase probably occurred due to the increasing number of vehicles. NO_2_ may be emitted from the transport and combustion of coal, but the emission originating from vehicles are relatively high [57]. NO_2_ also decreased during the study period (28% and 16% for summer and winter, respectively). The decrease in NO_2_ was not smaller than that in SO_2,_ and CO might be the relatively severe generation conditions of NO_2_ and its low proportion in the total NO_x_ emissions [58].

In contrast to other gaseous pollutants, O_3_ is influenced by many factors during formation, for examples, such as NO_x_, peroxyl radicals and volatile organic compounds (VOCs) [59]. In this study, O_3_ increased in both summer and winter from 2015 to 2017, especially in winter (from 14.88 ± 6.084 ppb to 27.71 ± 10.02 ppb). This may occur because the decrease in NO concentration due to motor vehicle emission reduction may lead to increasing ozone. The decrease in NO in the atmosphere may notably increase the concentration of ozone, and this situation has currently become one of the most worrying environmental problems in certain developed countries, such as Japan [59,60,61]. Additionally, the increasing amount of VOCs in China may increase the photochemical production of ozone, which may also be a reason [60]. The decrease in most gaseous pollutants reveals that the air pollution control policies implemented in Xinxiang City have achieved great success, especially the control policy of the reduction in coal combustion in winter. Although the regulations on vehicle exhaust emissions recently become increasingly strict, with the mounting number of vehicles and increasing traffic jams, the government of China should continue to maintain a positive attitude towards the control of gaseous pollutants.

### 3.5. Health Risk of PAHs and NPAHs

BaP_eq_ and ILCR value is often used to briefly speculated that the possible risk of the effect of PAHs and NPAHs on human health. Table 4 lists the mean and standard deviation (SD) of BaP_eq_ and ILCR. As shown in Table 4, BaP occupied the highest proportion in ΣBaPeq during all periods, followed by BbF. 2-NFR in NAPHs attained a high value in ΣBaPeq despite lower concentration than that of PAHs. This emphasizes the importance of NPAHs monitoring during the observation of the atmospheric environment.

The mean values of ΣBaP_eq_ in summer in 2015 and 2017 were 653 ± 140 pg/m^3^ and 736 ± 493 pg/m^3^, respectively. However, the values in winter in 2015 and 2017 were 10,140 ± 5835 pg/m^3^ and 5170 ± 4405 pg/m^3^, respectively. ΣBaP_eq_ in winter was approximately one order of magnitude higher than that in summer, and ΣBaP_eq_ in 2017 exhibited a dramatic decrease over the value in 2015. The dramatic decrease in BaP_eq_ in winter might be due to the effective implementation of the air pollution control policy in 2016, which decreased the emissions stemming from the thermal power industry and heating.

During the use of UR_BaP_ value of 1.1 × 10^−6^ ng/m^3^, the ILCR values in the winter of 2015 and 2017 were (11.2 ± 6.42) × 10^−6^ and (5.69 ± 4.85) × 10^−6^, respectively, which were significantly higher than those in summer [24]. Alternatively, by using the UR_BaP_ value of 8.7 × 10^−5^ ng/m^3^, the value can be up to (88.2 ± 50.8) × 10^−5^ and (45.0 ± 38.3) × 10^−5^ in the winter of 2015 and 2017, respectively. The value of the results significantly higher than those in summer as well. The results of ILCR by using of UR_BaP_ value of coke-oven workers were higher than the UR_BaP_ value of hamsters. Both ILCR with different UR_BaP_ values were higher than the acceptable standard (l × 10^−6^) defined by the US EPA [62].

The BaP_eq_ and ILCR value in this study makes a preliminary study of the health risks only. The results of BaPeq and ILCR in this study show relatively high health risks in Xinxiang, although the implementation of the policy has shown an improvement in the air quality. Further evaluation will be conducted to analyze the health risks in Xinxiang.

### 3.6. Research Limitations

In this study, some limitations occurred, which should be considered. Increasing the sampling periods and the collection and analysis, including gaseous PAHs, will enhance the reliability of the results obtained in this research, although this study has basically proved through the data of PAHs, NPAHs and the gaseous pollutants that the air pollution in Xinxiang has been improved a certain extent after the 2016 environmental protection policy. The publication of this article might also contribute to the consolidation and development of the policies.

## 4. Conclusions

As one of the most polluted cities in China, the air pollution issues and implementation effects of government policies in Xinxiang have received much attention. In this study, to evaluate the implementation effects of policies in Xinxiang, PAH and NPAH, data were collected in the summer and winter of 2015 and 2017. In general, both PAHs and NPAHs attained relatively high concentrations in winter. The concentration of PAHs exhibited a significant decrease in summer and winter from 2015 to 2017. Four-ring PAHs occupied a dominant position in summer and winter, indicating the high possibility of biomass and/or coal burning. In this study, NPAHs were mainly generated under the OH radical pathway and via secondary formation. Diagnostic ratios revealed that PAHs and NPAHs were largely affected by vehicle emissions in summer and coal and biomass emissions in addition to petroleum in winter. These results indicate that the effectiveness of the air pollution control policies implemented in Xinxiang had already achieved a certain preliminary effect. The ΣBaP_eq_ and ILCR exhibited an obvious downward trend from 2015 to 2017; the results indicated the health risk of cancer in the winter of Xinxiang should still remaining alert. Stringent air pollution control policies should still be implemented in Xinxiang city.

## Figures and Tables

**Table 1:** The mean concentration and the standard deviation of polycyclic aromatic hydrocarbons (PAH) and three nitro-PAHs (NPAH) in fine particulate matter (PM_2.5_) for 2015 to 2017.

Species	Ring Numbers	2015		2017	
		Summer	Winter	Summer	Winter
PAHs (ng/m^3^)					
FR	4	0.88 ± 0.13	19.8 ± 18.5	0.46 ± 0.17	11.7 ± 10.9
Pyr	4	1.41 ± 0.29	15.2 ± 12.4	0.36 ± 0.12	7.01 ± 6.46
BaA	4	0.28 ± 0.06	9.76 ± 8.36	0.27 ± 0.17	4.31 ± 3.70
Chr	4	0.48 ± 0.12	10.3 ± 6.87	0.55 ± 0.24	5.33 ± 4.12
BbF	5	1.31 ± 0.35	14.9 ± 9.31	1.09 ± 0.67	7.93 ± 7.07
BkF	5	0.29 ± 0.09	4.85 ± 2.95	0.29 ± 0.23	2.44 ± 2.25
BaP	5	0.38 ± 0.08	6.16 ± 3.24	0.47 ± 0.33	3.18 ± 2.68
BgPe	6	0.80 ± 0.20	8.70 ± 4.88	0.77 ± 0.43	4.35 ± 3.58
IDP	6	0.54 ± 0.12	7.27 ± 3.76	0.63 ± 0.37	3.49 ± 2.84
ƩPAHs		6.37 ± 1.30	96.9 ± 69.9	4.89 ± 2.67	49.8 ± 43.4
NPAHs (pg/m^3^)					
2-NFR	4	318 ± 74.0	1333 ± 559	420 ± 294	958 ± 896
2-NP	4	9.33 ± 4.83	240 ± 111	19.3 ± 12.2	163 ± 160
1-NP	4	8.27 ± 1.85	133 ± 87.5	17.5 ± 11.0	70.8 ± 59.4
ƩNPAHs		336 ± 77.2	1707 ± 708	456 ± 312	1192 ± 1113

**Table 2:** Correlations among the individual PAHs and NPAHs with meteorological conditions in PM_2.5_ during the sampling periods (*n* = 26).

Species	Temperature (℃)	Humidity (%)	Wind Level
FR	−0.78 **	−0.17	−0.26
Pyr	−0.75 **	−0.24	−0.25
BaA	−0.75 **	−0.01	−0.16
Chr	−0.72 **	0.13	−0.23
BbF	−0.75 **	−0.02	−0.17
BkF	−0.73 **	0.03	−0.18
BaP	−0.71 **	0.06	−0.15
BgPe	−0.72 **	0.02	−0.17
IDP	−0.68 **	0.09	−0.15
2-NFR	−0.40 *	0.14	−0.32
2-NP	−0.68 **	0.27	−0.31
1-NP	−0.64 **	0.16	−0.13

* *p* < 0.05, ** *p* < 0.01.

**Table 3:** The mean concentration and the standard deviation of gaseous pollutants during sampling (unit: ppb).

Species	2015		2017	
	Summer	Winter	Summer	Winter
SO_2_	15.66 ± 4.548	34.54 ± 8.740	4.826 ± 0.933	9.842 ± 6.321
CO	0.730 ± 0.170	3.297 ± 1.229	0.905 ± 0.272	1.734 ± 1.129
NO_2_	19.70 ± 3.503	45.12 ± 10.99	14.21 ± 1.653	37.84 ± 20.58
O_3_	74.81 ± 13.77	14.88 ± 6.084	82.61 ± 7.433	27.71 ± 10.02

**Table 4:** The mean benzo[*a*]pyrene (BaP)-equivalent concentrations (pg/m^3^), standard deviation (SD) and incremental lifetime cancer risk (ILCR) of PAHs and NPAHs in 2015 and 2017.

Species	2015		2017	
	Summer	Winter	Summer	Winter
FR	0.88 ± 0.13	19.8 ± 18.5	0.46 ± 0.17	11.7 ± 10.9
Pyr	1.41 ± 0.29	15.2 ± 12.4	0.36 ± 0.12	7.01 ± 6.46
BaA	28.1 ± 5.63	976 ± 836	27.5 ± 16.6	431 ± 370
Chr	4.79 ± 1.19	103 ± 68.7	5.50 ± 2.44	53.3 ± 41.2
BbF	130 ± 34.7	1485 ± 931	109 ± 66.6	793 ± 707
BkF	28.2 ± 8.99	485 ± 295	29.3 ± 22.7	244 ± 225
BaP	379 ± 79.5	6162 ± 3239	471 ± 332	3182 ± 2681
BgPe	8.01 ± 2.02	87.0 ± 48.8	7.65 ± 4.30	43.5 ± 35.8
IDP	54.4 ± 11.6	727 ± 376	62.5 ± 37.3	349 ± 284
2-NFR	15.9 ± 3.70	66.7 ± 28.0	21.0 ± 14.7	47.9 ± 44.8
1-NP	0.83 ± 0.19	13.4 ± 8.75	1.75 ± 1.10	7.08 ± 5.94
Total BaP_eq_	653 ± 140	10140 ± 5835	736 ± 493	5170 ± 4405
ILCR (hamsters)	(0.72 ± 0.15) × 10^−6^	(11.2 ± 6.42) × 10^−6^	(0.89 ± 0.54) × 10^−6^	(5.69 ± 4.85) × 10^−6^
ILCR (coke-oven workers)	(5.69 ± 1.21) × 10^−5^	(88.2 ± 50.8) × 10^−5^	(6.40 ± 4.29) × 10^−5^	(45.0 ± 38.3) × 10^−5^

## Data Availability

The data presented in this study are available in the Supplementary material.

## Supplementary Materials

The following are available online at https://www.mdpi.com/1660-4601/18/6/3017/s1, Table S1: Meteorological conditions of Xinxiang during sampling. Table S2: Limit of determination (LOD) and the range of calibration curves of PAHs and NPAHs, Table S3: The TEF value of each PAH and NPAH, Table S4: Daily concentration of PAHs and NPAHs of PM_2.5_ in summer and winter during 2015–2017. Table S5: Seasonal correlations among the individual PAHs and NPAHs with meteorological conditions in PM_2.5_ during the sampling periods (*n* = 13).
