# Supplementary material for: Characteristics and Health Risks of Polycyclic Aromatic Hydrocarbons and Nitro-PAHs in Xinxiang, China in 2015 and 2017
Source: Int J Environ Res Public Health. 2021 Mar 15;18(6):3017. doi: 10.3390/ijerph18063017 (PMC8002061; doi:10.3390/ijerph18063017)
Supplement: Supplementary file 1 [file ijerph-18-03017-s001.pdf]

Supplementary Materials

Table 1. Meteorological conditions of Xinxiang during sampling.

Meteorological Conditions	2015		2017	
	Summer	Winter	Summer	Winter
Temperature (℃)	27.1 ± 2.2	2.2 ± 2.3	28.6 ± 2.1	0.5 ± 1.8
Humidity (%)	40.3 ± 11.9	60.4 ± 11.3	72.5 ± 3.9	58.1 ± 15.6
Wind level	1.5 ± 0.5	1.3 ± 0.6	1.5 ± 0.6	1.2 ± 0.4

Wind Level: Beaufort wind force scale, level 1 = 0.3 – 1.5 m/s; level 2 = 1.6–3.3 m/s etc.

Table 2. Limit of determination (LOD) and the range of calibration curves of PAHs and NPAHs.

Species	LOD (pg/mL)	Range of calibration curves (ng/mL)
FR	16.2	0.2–199
Pyr	30.3	1.0–99
BaA	9.1	0.1–99
Chr	34.3	1.0–99
BbF	55.5	1.0–200
BkF	8.8	0.1–100
BaP	8.8	0.1–100
BgPe	55.3	1.0–199
IDP	82.9	1.0–99
1-NP	12.4	0.12–124
2-NP	6.2	0.12–24.7
2-NFR	5.0	0.06–24.7

Table 3. The TEF value of each PAH and NPAH [21-22].

PAH and NPAH	TEF_i
FR	0.001
Pyr	0.001
BaA	0.1
Chr	0.01
BbF	0.1
BkF	0.1
BaP	1
BgPe	0.01
IDP	0.1
2-NFR	0.05
2-NP	/ [a]
1-NP	0.1

[a] = No TEF value.

Table S4. Daily concentration of PAHs and NPAHs of $PM_{2.5}$ in summer and winter from 2015 to 2017.

Periods	Sampling Date	PAHs (pg/m³)										NPAHs (pg/m³)			
		FR	Pyr	BaA	Chr	BbF	BkF	BaP	BgPe	IDP	ΣPAHs	2-NFR	2-NP	1-NP	ΣNPAHs
2015-Summer	2015/6/3	802	1320	247	436	1166	226	314	643	442	5597	273	4.93	6.06	283
	2015/6/5	977	1170	367	572	1472	365	483	907	632	6947	339	7.00	10.4	356
	2015/6/8	867	1389	252	386	1019	262	391	780	551	5897	304	7.97	7.86	320
	2015/6/10	749	1268	226	335	957	178	286	557	382	4939	248	6.09	7.72	262
	2015/6/12	1082	1992	334	653	1896	422	459	1128	695	8661	456	12.3	10.7	479
	2015/6/15	784	1328	256	493	1339	297	342	790	562	6192	292	17.7	6.96	317
	Average	877	1411	281	479	1308	292	379	801	544	6372	319	9.33	8.27	336

2017-Summer	2017/7/13	269	232	70.6	158	295	48.7	111	265	199	1647	96.1	3.11	4.61	104
	2017/7/15	463	453	387	701	1376	396	638	943	781	6138	933	29.4	21.2	984
	2017/7/17	402	278	179	463	646	134	231	458	379	3171	351	13.6	9.14	373
	2017/7/19	317	258	143	504	502	73.5	154	340	236	2528	270	9.86	9.34	289
	2017/7/21	427	313	203	392	986	240	397	774	625	4356	179	14.3	17.6	211
	2017/7/23	781	494	434	795	1798	555	861	1241	1026	7983	433	28.4	24.4	486
	2017/7/25	542	519	508	842	2036	602	907	1334	1131	8419	675	36.7	36.4	749
	Average	457	364	275	550	1091	293	471	765	625	4892	420	19.3	17.5	456
2015-winter	2015/12/26	9618	8552	4830	5269	9058	2653	3526	5119	3995	52619	424	60.3	74.9	559
	2015/12/28	13490	10365	6890	8322	11480	3829	4969	6855	5965	72166	1000	207	105	1313
	2015/12/30	12042	9766	6049	7606	11064	3693	5019	6865	5666	67770	1664	188	92.4	1945
	2016/1/1	11110	9285	5542	9490	11737	3973	5316	7649	7141	71241	1784	313	99.1	2196
	2016/1/3	57294	40242	26615	24029	33727	10777	12617	18499	14655	238455	1888	368	310	2566
	2016/1/5	15388	12718	8632	7109	12028	4189	5525	7189	6201	78980	1240	307	121	1668
	Average	19824	15155	9760	10304	14849	4852	6162	8696	7270	96872	1334	241	134	1708
2017-winter	2018/1/5	3499	1980	1446	2434	3093	921	1110	1916	1546	17945	281	63.4	31.0	376
	2018/1/7	3256	1484	1111	1570	2352	574	816	1330	1005	13498	158	20.6	25.6	205
	2018/1/9	3251	2123	1558	1881	2656	682	1090	1400	1019	15660	230	17.5	27.1	275
	2018/1/11	5241	4063	2532	2699	3553	1094	1868	2365	2133	25549	463	78.9	32.1	574
	2018/1/13	13433	7432	4625	7484	8090	2697	3718	4909	3992	56380	1289	202	70.7	1562
	2018/1/15	26522	15974	9864	10472	17139	5366	6575	8853	7131	107896	2260	359	147	2767
	2018/1/17	27007	16045	9015	10745	18600	5767	7100	9708	7616	111602	2027	398	162	2587
	Average	11744	7014	4307	5326	7926	2443	3182	4355	3492	49790	958	163	70.8	1192

Table S5. Seasonal correlations among the individual PAHs and NPAHs with meteorological conditions in $PM_{2.5}$ during the sampling periods ($n = 13$).

Species	Summer			Winter		
	Temperature (℃)	Humidity (%)	Wind Level	Temperature (℃)	Humidity (%)	Wind Level
FR	−0.553	−0.624*	0.194	0.337	0.518	−0.353
Pyr	−0.615*	−0.721**	0.103	0.521	0.353	−0.242
BaA	−0.451	0.006	0.515	0.521	0.344	−0.242
Chr	−0.230	0.347	0.320	0.521	0.543	−0.343
BbF	−0.462	−0.094	0.509	0.536	0.433	−0.289
BkF	−0.371	0.022	0.492	0.487	0.468	−0.343
BaP	−0.292	0.127	0.623*	0.507	0.455	−0.343
BgPe	−0.371	0.022	0.492	0.587*	0.438	−0.289
IDP	−0.315	0.132	0.595*	0.598*	0.441	−0.289
2-NFR	−0.473	0.201	0.189	0.419	0.488	−0.471
2-NP	−0.184	0.556*	0.229	0.315	0.584*	−0.498
1-NP	0.096	0.424	0.538	0.504	0.433	−0.289

* $p < 0.05$, ** $p < 0.01$.
